# Three-Dimensional Soft Material Micropatterning via Grayscale Photolithography for Improved Hydrophobicity of Polydimethylsiloxane (PDMS)

**DOI:** 10.3390/mi13010078

**Published:** 2022-01-01

**Authors:** Intan Sue Liana Abdul Hamid, Beh Khi Khim, Mohammad Faiz Mohamed Omar, Khairu Anuar Mohamad Zain, Nuha Abd Rhaffor, Sofiyah Sal Hamid, Asrulnizam Abd Manaf

**Affiliations:** 1 Faculty of Electrical and Electronic Engineering, Universiti Tun Hussein Onn Malaysia, Batu Pahat 86400, Johor, Malaysia; liana@uthm.edu.my; 2 Microelectronic & Nanotechnology—Shamsuddin Research Centre (MiNT-SRC), Universiti Tun Hussein Onn Malaysia, Batu Pahat 86400, Johor, Malaysia; 3 Collaborative Microelectronic Design Excellence Center (CEDEC), Sains@USM, Universiti Sains Malaysia, Bayan Lepas 11900, Pulau Pinang, Malaysia; kkbeh@usm.my (B.K.K.); faiz_omar@usm.my (M.F.M.O.); anuar@usm.my (K.A.M.Z.); nuha@usm.my (N.A.R.); sofiyah@usm.my (S.S.H.)

**Keywords:** grayscale lithography, hydrophobic, micro 3D structure, droplet, contact angle

## Abstract

In this present work, we aim to improve the hydrophobicity of a polydimethylsiloxane (PDMS) surface. Various heights of 3D PDMS micropillars were fabricated via grayscale photolithography, and improved wettability was investigated. Two approaches of PDMS replication were demonstrated, both using a single master mold to obtain the micropillar arrays. The different heights of fabricated PDMS micropillars were characterized by scanning electron microscopy (SEM) and a surface profiler. The surface hydrophobicity was characterized by measuring the water contact angles. The fabrication of PDMS micropillar arrays was shown to be effective in modifying the contact angles of pure water droplets with the highest 157.3-degree water contact angle achieved by implementing a single mask grayscale lithography technique.

## 1. Introduction

A solid surface’s hydrophobicity is the physical property that makes it water repellent and non-wettable. In microfluidic applications, super hydrophobic materials have a significant impact. In the channel walls of microfluidic chips, super hydrophobic surfaces are preferred since the water-repellent surfaces will reduce the pressure losses of liquid flow [1]. Open microfluidics is yet another important use. As there is no continuous flow in an open microfluidic application, super hydrophobic surfaces are required to form water droplets. The droplet will be held by the surfaces with the sensor underneath, which can execute a range of fluidic functions such as mixing, merging, dispersing, splitting, and transporting [2,3]. Moreover, a pipetting-free droplet array can be created simply on the polydimethylsiloxane (PDMS) super hydrophobic surface. In comparison to the existing method, which involves manual pipetting on 96- or 384-well microplates, this could be a viable alternative for generating droplet arrays [4,5]. With the ability to change the composition of each droplet, a huge number of experiments can be run simultaneously. Thus, the fabrication technique of a micro three-dimensional (3D) structure on a PDMS surface with a high-aspect ratio (HAR) is one of the alternatives to realize a PDMS-based pipetting-free droplet array. The conventional photolithography technique is only capable of fabricating low aspect ratio structures [6,7,8]. A modification in conventional photolithography to fabricate high aspect ratio structures with a standard binary mask is demonstrated in order to achieve a reliable fabrication method for microfluidic devices [9]. However, it has led to a rise in fabrication process complexity. Multiple masks must be prepared and multiple ultraviolet (UV) exposures are needed to fabricate many phase levels of microstructures. Multiple mask fabrication and exposure will increase the alignment error, which leads to an increase in production time.

The 3D microstructures fabricated as an array of micropillars may help to increase the surface roughness and change the hydrophobicity of the surface. The hydrophobicity of a surface is determined by measuring the water contact angle on the surface. The grayscale photolithography process has been proposed in this research to overcome the limitation of conventional photolithography to fabricate 3D and high-aspect ratio microstructures with a high contact angle in a superhydrophobic wetting state. This paper demonstrates an implementation of grayscale photolithography with a single mask and single exposure capable of fabricating microstructures with different HAR structures to overcome the problems encountered with conventional photolithography and 3D printing techniques.

## 2. Background of Hydrophobic Properties

Super hydrophobic surfaces have water contact angles of more than 150 degrees and can be achieved by increasing surface roughness while keeping the surface energy low [10]. Wenzel and Cassie–Baxter models describe the effect of surface roughness on wettability. With increasing surface roughness in both models, the effective water contact angle increases [11,12]. In the Wenzel condition, the water droplet is completely in contact with the rough surface. In the Cassie–Baxter condition, however, the water droplet is positioned on top of the rough surface, with a trapped air pocket beneath the water droplet.

The purpose of this research is to improve the surface roughness of hydrophobic materials by implementing a grayscale lithography technique with a single exposure process. Because it is initially hydrophobic, PDMS is an excellent example of such materials. The fabrication of well-arranged microstructures, such as micropillars, is used to create PDMS super hydrophobic surfaces. The photolithography process can manufacture well-defined structures with a uniform shape and dimension, which is ideal for generating super hydrophobic PDMS. Various studies have been conducted on surface wettability modification through the photolithography technique [13,14,15]. However, by an employing the conventional photolithography technique, the hydrophobicity of the surface was increased but did not achieve the superhydrophobic wetting state with a maximum contact angle measured as 140.4${}^{{}^{\circ}}$ [14]. In this paper, we aim to investigate the impact of grayscale photolithography on fabricating various heights of 3D micropillars, simultaneously using only a one-layer mask in improving the hydrophobicity of the PDMS surface towards being superhydrophobic. Finally, the surface hydrophobicity is characterized by measuring the water contact angles.

## 3. Materials and Method

### 3.1. Materials

High precision photo plate emulsion masks and the emulsion mask developer (CDH-100) solution were supplied by Konica Minolta Inc. (Tokyo, Japan) The microscope glass slides were supplied by DURAN group (Huntington Beach, CA, USA). Acetone (C${}_{3}$H${}_{6}$O), methanol (CH${}_{3}$OH), and iso-2-Propanol (C${}_{3}$H${}_{8}$O) were all provided by J.T. Baker, US. Polydimethylsiloxane (PDMS) was purchased from Sigma-Aldrich (Burlington, MA, USA), and SU8-10 photoresist was purchased from MicroChem (Westborough, MA, USA). All the chemicals were used as received, and distilled water was used for all experiments.

### 3.2. Development of PDMS Surface Wettability Modification

#### 3.2.1. Design Parameter of Array of Microstructure

**Figure 1:**
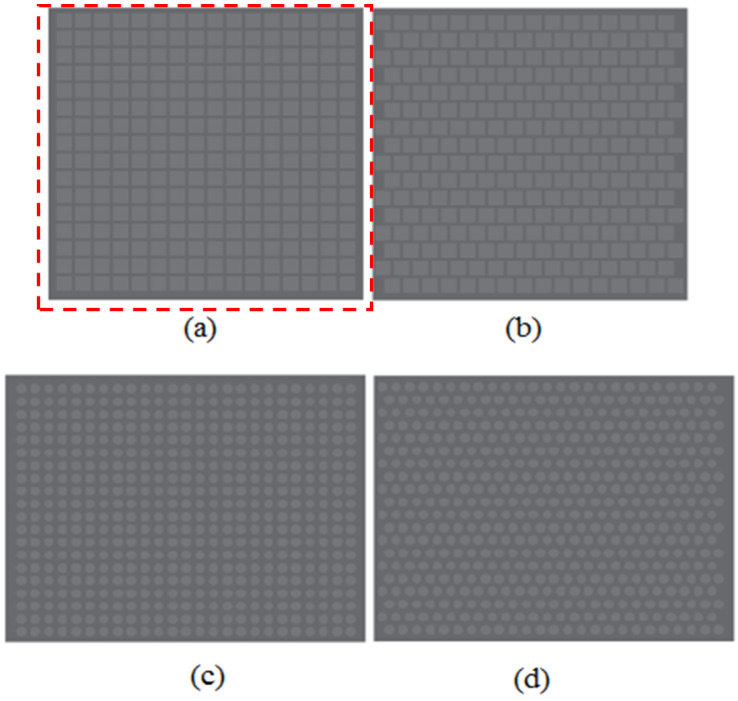
PET mask designs of (**a**) square cross sectional with square distribution, (**b**) square cross sectional with hexagonal distribution, (**c**) circle cross sectional with square distribution, and (**d**) circle cross sectional with hexagonal distribution.

**Figure 2:**
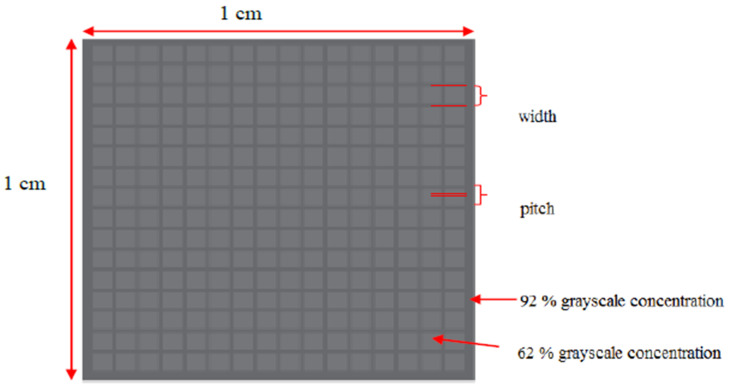
Zoomed in software-designed mask design parameters.

Figure 1 describes the software-designed mask design parameters. The micropillars were designed in square cross and circle cross sectional shapes with 200 μm, 100 μm, and 80 μm width as shown in Figure 1. The pitch value 100 μm and the pillar height was determined by the grayscale concentration of 60% as shown in Figure 2. The pitch and grayscale were unchanged for all types of width design. The width dimensions of the micropillars were chosen with respect to the height obtained by the 60% grayscale concentration. The relationship between pillar width and height is given by the aspect ratio values, where the aspect ratio must be between 0.2 and 2 to avoid the deformation of the PDMS micropillar [16]. Figure 1 shows the preliminary PET mask design of the micropillar array for square shape and circle shape. The microhole has a square cross section and circle cross section with width values of 80 μm, 100 μm, and 200 μm, and the array was arranged in square and hexagonal distribution.

#### 3.2.2. Grayscale Mold Fabrication Method

**Figure 3:**
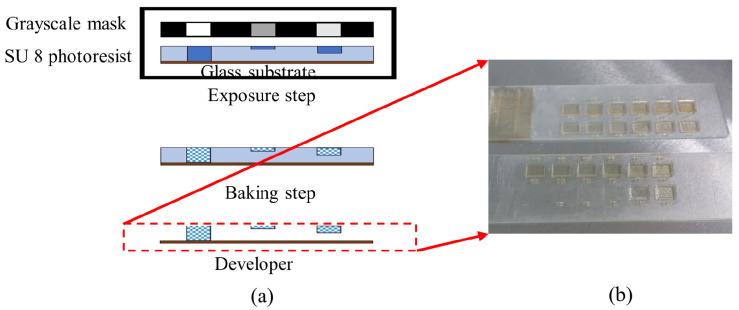
(**a**) Grayscale fabrication method and (**b**) real image of SU8 mold.

**Figure 4:**
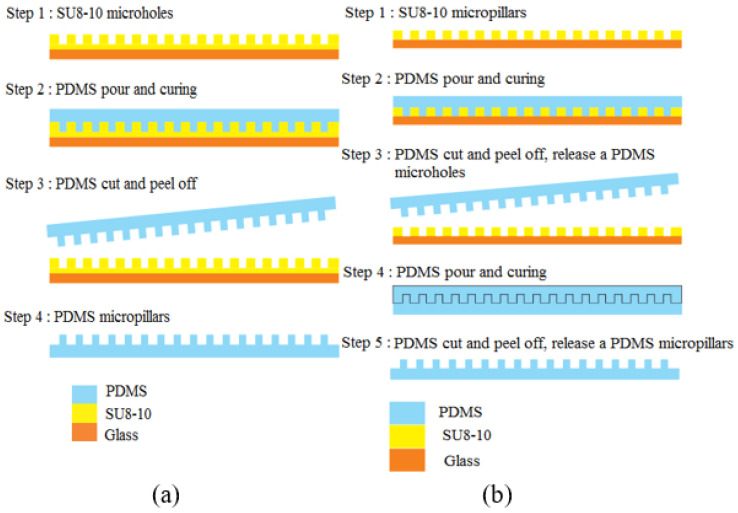
Fabrication steps of SU8 micropillar using (**a**) SU8-10 microhole mold, and (**b**) PDMS microhole mold (PDMS–PDMS pattern transfer).

The process begins with the emulsion mask (High Precision Photo Plate from Konica Minolta Inc.) preparation. Emulsion mask was prepared by optical projection lithography using software-designed mask as the master mask. The software-designed mask was printed on a transparent based polyethylene terephthalate (PET) film. Then, the image on the software mask was transferred onto emulsion mask by using the Simple Mask Fabrication Machine (MM605 from Nanometric Technology Inc. (Tokyo, Japan)) in 5 to 1 scaling-down ratio. After that, the thick SU8 coat on microscope glass slide sample was prepared by dispensing 2.5 mL volume of SU8-10 on a clean 3-inch × 1-inch microscope glass slide from DURAN group. The coated glass substrate goes through a soft baking process at 95 °C for 7 h on a hotplate. Then, the coated glass substrate was aligned with the emulsion mask for UV light exposure. The exposure process was carried out using One Side Mask Aligner (LA4100_R1 Sanei Electric Inc. (Tokyo, Japan)) with power density 180 W of the i-line mercury lamp. The photoresist SU8-10 was exposed for 60 s by back side exposure method. The samples were baked at 65 °C for 2 min and then at 95 °C for 10 min. The samples were cooled down gradually, with 5 °C interval of temperature reduction until it achieved room temperature. Finally, the samples go through development process using SU8 developer to obtain the micro mold pattern as shown in Figure 3. The micro mold was then replicated by PDMS mixture. PDMS mixture was prepared with 10 to 1 weight ratio of base polymer and hardener solution. The mold was placed in an aluminum foil boat, and then the PDMS mixture was poured on top of it. The PDMS heat curing hardened and replicated the pattern on micro mold as shown in Figure 4a.

#### 3.2.3. SU8-10 Mold Fabrication

Firstly, the SU8 mold having the microhole array structure was fabricated through grayscale fabrication technique as shown in Figure 1. The size of patterned area was 10 mm × 10 mm as shown in Figure 2. The percentage of grayscale concentration on emulsion mask will affect the percentage of UV penetration through grayscale mask. Thus, it will determine the height of the replicated PDMS micropillar array for SU8 mold as indicated in our study in [17]. The image in Figure 3 denotes the SU-8 10 structure after development process. The difference in SU-8 10 thickness was associated with the percentage of grayscale concentration. The different grayscale concentrations of the patterned emulsion mask will allow UV dose gradient to form latent image during exposure. Even though the SU8-10 is exposed by a uniform UV exposure dose, the cross-linked SU8-10 will solidify in structures of different heights according to their respective grayscale concentration.

The percentages of grayscale concentration were chosen with reference to our previous work on grayscale photolithography process development [17]. The SU8-10 mold has been designed using two different depths presented by two different values of grayscale concentration. The 62% grayscale concentration was chosen for the square microstructures, and 92% grayscale concentration for the border between the squares. From our previous study in [17], the corresponding SU8-10 thickness is 1003.7 μm for 92% and 478.2 μm for 62% grayscale concentration. The depth of the square microstructure is defined by the difference between thickness of the border and the square microstructures and given as 525.5 μm. The design was repeated for several sizes of the square microstructures and was observed for the effect on surface wettability.

#### 3.2.4. Double Stamping PDMS Relief Fabrication Method

**Figure 5:**
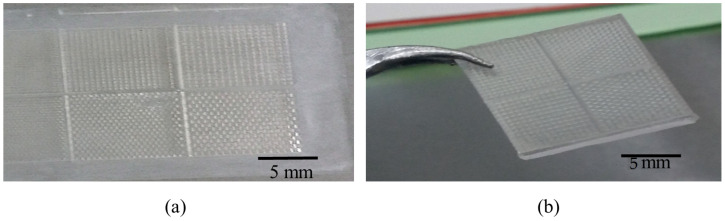
(**a**) Image of array of SU8 mold and (**b**) image of PDMS relief.

Another SU8 mold having the micropillar array structure with the same dimensions was fabricated and replicated by PDMS with heat curing at 120 °C for 1 h. The resulting PDMS microhole array was then replicated by PDMS and cured at room temperature. The PDMS–PDMS pattern transfer results in PDMS micropillar array structure. The PDMS–PDMS pattern transfer process is illustrated in Figure 4b. To evaluate the fabricated PDMS micropillars, the samples were observed using a scanning electron microscope (SEM) and surface profiler. The image of array of micro pillar SU8 mold and PDMS relief is shown in Figure 5a,b, respectively.

### 3.3. Water Contact Angle Measurement

**Figure 6:**
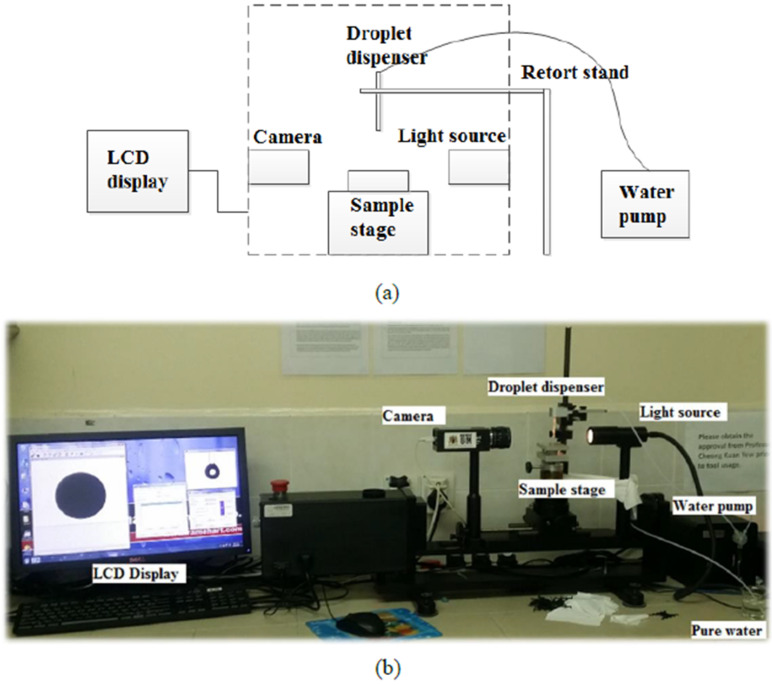
Contact angle measurement setup: (**a**) schematic diagram and (**b**) image of actual measurement setup.

The changes in PDMS surface roughness were evaluated by measuring the water contact angle. Water contact angle is determined by using the Goniometer (Rame-hart Instrument. Co., Succasunna, NJ, USA) with room temperature environment. The PDMS micropillar array structure was placed on the sample stage, and three distinct sections of the surface were repeatedly dispensed with pure water droplets of 3 μL volume. The contact angle value was determined using the instrument’s software and shown on the computer monitor. Figure 6 describes the contact angle measurement experiment setup.

## 4. Results and Discussion

### 4.1. Hydrophobic PDMS

**Figure 7:**
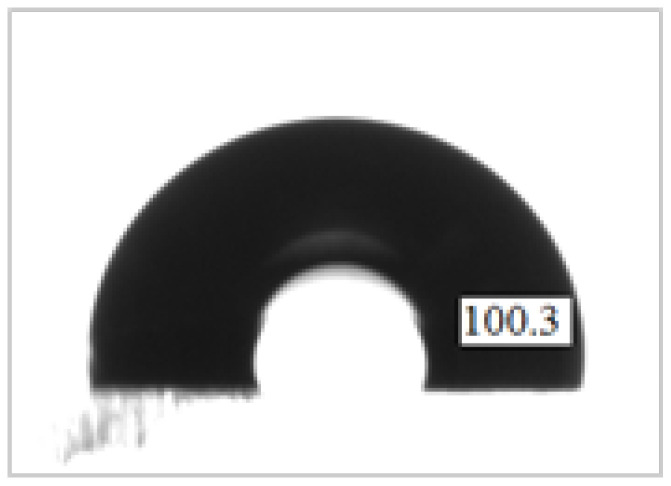
Water contact angle of a plane PDMS.

**Figure 8:**
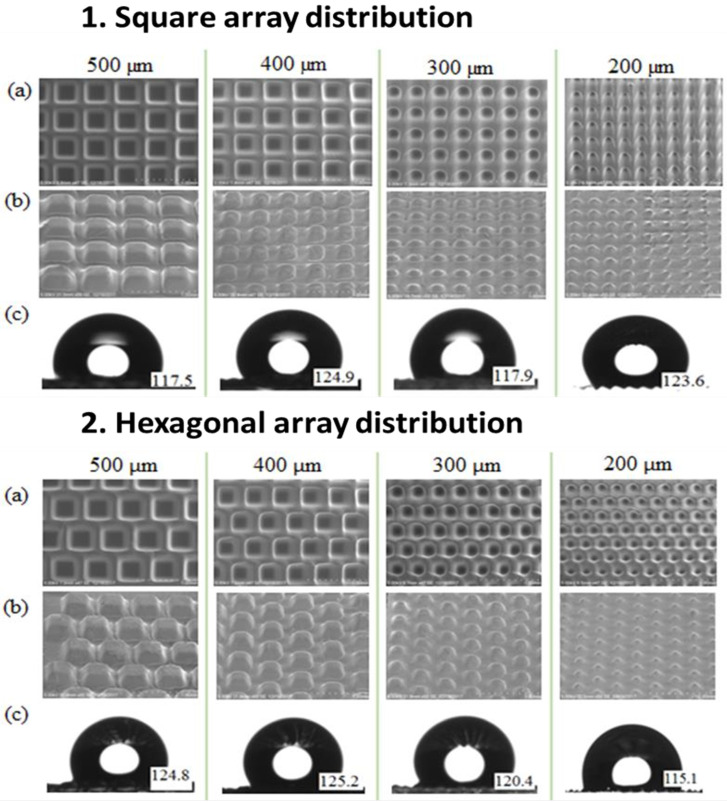
Top view SEM images of: (**a**) SU8 microhole mold, (**b**) replicated PDMS micropillar, and (**c**) water contact angle value for square and hexagonal array distribution.

In the first experiment, we fabricate the PDMS micropillar array by replicating the microhole pattern on the SU8 mold, as described in Figure 4a. The contact angle of the plain PDMS surface was measured. The contact angle was 100.3 as shown in Figure 7. Figure 8a shows the SEM images of the SU8 microhole mold for the square pillar cross section of 500 μm, 400 μm, 300 μm, and 200 μm width. The measured widths of the fabricated pillar cross section are 501 μm, 369 μm, 286 μm, and 194 μm. The image quality degradation has been observed to be associated with line shortening and corner rounding. Figure 8b shows the SEM images of the replicated PDMS micropillar from the SU8 microhole mold. Using the surface profiler, the heights of the replicated PDMS micropillar for the square pillar cross section of 200 μm, 300 μm, 400 μm, and 500 μm were measured as 32.8 μm, 47.3 μm, 108.7 μm, and 101.1 μm. The water droplet images on the micropillar arrays with the measured water contact angles are described in Figure 8c. The contact angle on a plane PDMS surface is recorded as 100.3 degrees. The fabrication of the PDMS micropillar array by using the SU8 microhole mold increased the contact angle of the PDMS surface (from 115.1 degrees to 125.5 degrees) when compared to the plane PDMS surface. Even though the hydrophobicity of the PDMS was increased, the wetting state was maintained as hydrophobic. The surface is considered to be super hydrophobic when the contact angle is measured as greater than 150 degrees.

### 4.2. Super Hydrophobic PDMS

**Figure 9:**
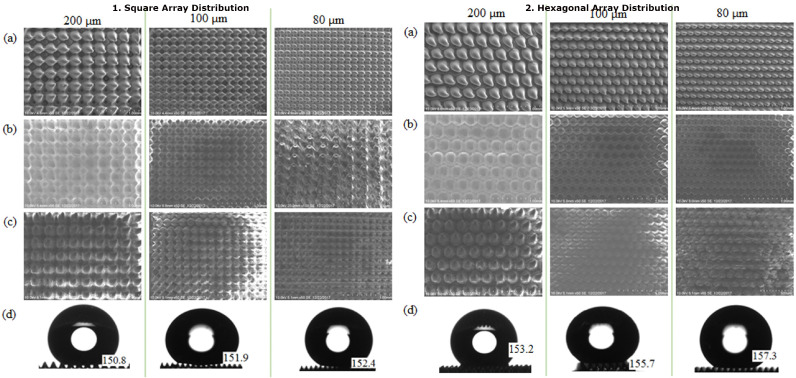
SEM images of: (**a**) SU8 micropillar, (**b**) PDMS microhole, (**c**) PDMS micropillar, and (**d**) water contact angle values.

**Figure 10:**
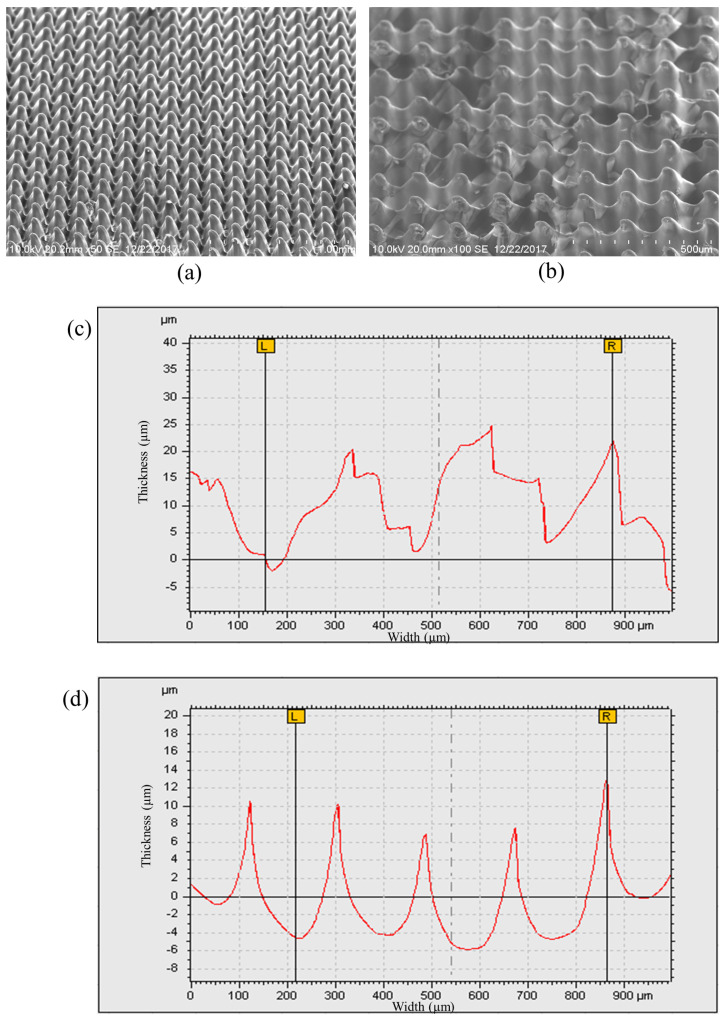
Micropillars with 80 μm width: (**a**) SU8-10 micropillar master mold, (**b**) replicated PDMS micropillar, (**c**) surface profile of the SU8-10 micropillar master mold, and (**d**) surface profile of the replicated PDMS micropillar.

**Figure 11:**
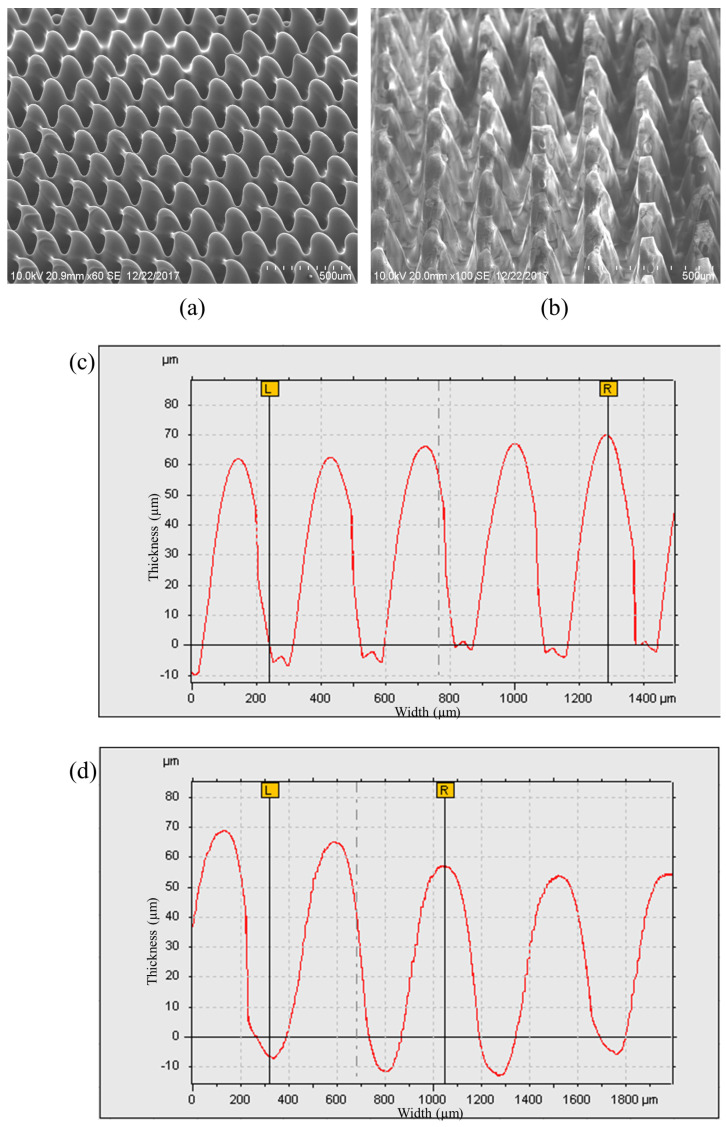
Micropillars with 200 μm width: (**a**) SU8-10 micropillar master mold, (**b**) replicated PDMS micropillar, (**c**) surface profile of the SU8-10 micropillar master mold, and (**d**) surface profile of the replicated PDMS micropillar.

Many researchers have used the SU8-10 microhole mold to fabricate PDMS micropillars in their studies, as mentioned in Section 3.2 [7,18,19,20]. We carried out the second experiment with the fabrication of the PDMS micropillar array by replicating the microhole pattern on the PDMS mold which is also referred to as PDMS–PDMS pattern transfer. The PDMS mold with the microhole pattern was prepared by the same PDMS replication method by using the SU8 micropillar mold. The detailed steps of fabrication are listed in Figure 4b. Figure 9a shows the SEM images of the SU8 micropillar mold with a smaller square pillar cross section of 200 μm, 100 μm, and 80 μm width. The wider bottom and narrower top of micropillars have been observed. This is the result of the back exposure method used in combination with the grayscale fabrication technique. In the back exposure method, the mask and SU8 were separated by a glass substrate. In addition, the thick SU8 coating results in a non-uniform light penetration through the SU8 with over exposed SU8 near the glass substrate and under exposed SU8 far off the glass substrate. Figure 9b shows the SEM images of the PDMS microhole replicated from the SU8 micropillar mold. Then, the PDMS–PDMS pattern transfer process was carried out, and SEM images of the resulting PDMS micropillar from the PDMS microhole mold replication are shown in Figure 9c. The water droplet images on the micropillar arrays with the measured water contact angles are described in Figure 9d. Figure 10 and Figure 11 show the detailed measurement of micropillar width of 80 μm and 200 μm, respectively. The measurements of the SU8 micropillar mold and PDMS micropillar height were carried out by using a surface profiler and are summarized in Table 1.

**Figure 12:**
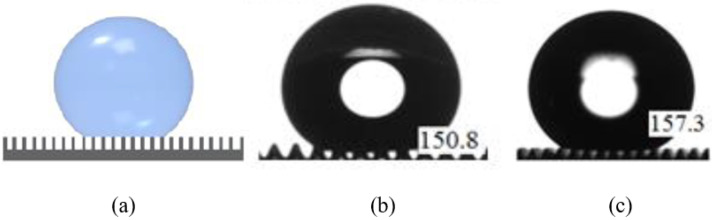
Cassie–Baxter super hydrophobic wetting state of the PDMS surface; (**a**) schematic, (**b**) actual measurement (minimum 150.8 degrees), and (**c**) actual measurement (maximum 157.3 degrees).

The heights of the SU8 micropillar mold were designed with the same grayscale concentration. However, from Table 1, the measured heights are not uniform. The resulting heights were affected by the diffraction effect due to the backside exposure method. The smaller size of the micropillar square cross section pattern leads to weak crosslinking within the SU8 during UV exposure due to the diffraction effect. After the development process, the smaller pattern-sized micropillar will be developed with a smaller height value [21]. The replicated PDMS micropillars have reduced height as compared to the original mold. This is due to the hydrophobicity of the PDMS. PDMS has a low surface energy, and this makes it hard to fill up the mold cavity and replicate the actual height of the mold. This PDMS–PDMS pattern transfer method using the SU8 microhole mold as the initial master mold attained the Cassie–Baxter super hydrophobic wetting state of the PDMS surface with contact angle values greater than 150 degrees (from 150.8 degrees to 157.3 degrees) as shown in Figure 12. In the Cassie–Baxter super hydrophobic state, the liquid droplet is only in contact with the tips of the PDMS micropillars [22]. The advantage of using this method is that the use of a single master mold can be maintained.

The 200 μm, 100 μm, and 80 μm micropillars in a hexagonal distribution also achieved superhydrophobicity with the Cassie–Baxter wetting state, with the highest contact angle recorded as 157.3°. Equation (1) was used to quantitatively evaluate the contact angle values of water on the fabricated PDMS micropillars. The Cassie–Baxter equation is given as

(1) $$\cos\theta_{r}=f\cos\theta_{s}-\left(1-f\right)$$

where $\theta_{r}$ is the contact angle of a liquid droplet placed on a textured surface, $\theta_{s}$ is the contact angle of a liquid droplet placed on a smooth surface, and *f* is the fraction of the surface that has contact with the liquid droplet. The $\theta_{s}$ is the value of the contact angle at a flat surface of PDMS. The contact angle is measured as 100.3${}^{{}^{\circ}}$, as shown in Figure 7. In the superhydrophobic state, the liquid droplet is assumed to be only in contact with the tips of the PDMS micropillars. From the observation of the PDMS micropillars in Figure 9c, the circle area of the pillar tip and a square area of a unit cell of the micropillar pattern were in contact with the liquid droplet. For a pillar width of 200 μm, the value of *f* is 0.0154, giving a value for the contact angle of 170.9${}^{{}^{\circ}}$. Meanwhile, for a pillar width of 100 μm and 80 μm, the value of f is 0.1018 and 0.0314, giving the values for the contact angle of 156.4${}^{{}^{\circ}}$ and 166.9${}^{{}^{\circ}}$, respectively. The calculations are listed in Table 2. The calculated contact angle values have proven that the fabricated PDMS micropillars increase the hydrophobicity of the PDMS into the superhydrophobic wetting state with a contact angle exceeding 150${}^{{}^{\circ}}$. To summarize, all superhydrophobic PDMS micropillar designs are listed in Table 2.

## 5. Conclusions

We have successfully demonstrated the improved hydrophobicity of a PDMS surface by changing its surface roughness using a single mask grayscale lithography technique. The PDMS surface roughness was increased by fabricating arrays of micropillars on the surface. In this work, first, we fabricated the SU8 microhole mold to attain the replicated PDMS micropillar array. With the PDMS–PDMS pattern transfer technique at a 200 μm width, the PDMS surface wettability has been increased into a super hydrophobic state with the highest contact angle of 157.3 degrees. The use of a single master mold can be maintained, but this time, we started the fabrication with the SU8 micropillar mold. A parallel comparison of the water contact angle measured in the second experiment with the first experiment of the same 200 μm width of a single micropillar with a square cross section has proved that PDMS–PDMS pattern transfer is the better solution for improving the hydrophobicity of a PDMS surface. As a conclusion, grayscale photolithography has been proven to fabricate a super hydrophobic PDMS surface with the potential to be used in open microfluidics in the future.

## Figures and Tables

**Table 1:** SU8 micropillar mold; PDMS micropillar measured by surface profiler.

Pillar Width (μm)	SU8-10 Micropillar Height (μm)	PDMS Micropillar Height (μm)
80	22.53	17.31
100	47.04	45.39
200	72.24	63.33

**Table 2:** The calculation of water contact angle on a rough surface.

Pillar Width (μm)	Fraction, *f* = $\frac{\mathrm{area}\;\mathrm{of}\;a\;\mathrm{single}\;\mathrm{pillar}}{\mathrm{area}\;\mathrm{of}\;a\;\mathrm{unit}\;\mathrm{cell}\;\mathrm{of}\;\mathrm{the}\;\mathrm{pattern}}$	Contact Angle of Rough Surface, $\theta_{r}$ $\cos\theta_{r}=f\cos\theta_{s}-\left(1-f\right)$
80	$f=\frac{\pi j^{2}}{80+100}=\frac{\pi \left(18^{2}\right)}{180}=0.0314$	$\cos\theta_{r}=-0.9742$
		$\theta_{r}=166.96^{{}^{\circ}}$
100	$f=\frac{\pi j^{2}}{100+100}=\frac{\pi \left(36^{2}\right)}{200}=0.1018$	$\cos\theta_{r}=-0.9164$
		$\theta_{r}=156.41^{{}^{\circ}}$
200	$f=\frac{\pi j^{2}}{200+100}=\frac{\pi \left(21^{2}\right)}{300}=0.0154$	$\cos\theta_{r}=-0.9874$
		$\theta_{r}=170.88^{{}^{\circ}}$
